# A scoping review of the discrepancies in the measurement of cerebral blood flow in idiopathic intracranial hypertension: oligemia, euvolemia or hyperemia?

**DOI:** 10.1186/s12987-023-00465-w

**Published:** 2023-08-23

**Authors:** Grant A Bateman

**Affiliations:** 1 Department of Medical Imaging, John Hunter Hospital, Locked Bag 1, Newcastle Region Mail Center, Newcastle, NSW 2310 Australia; 2 Newcastle University Faculty of Health, Callaghan Campus, Newcastle, NSW Australia

**Keywords:** Anemia, Cerebral blood flow, Idiopathic intracranial hypertension, Obesity, Stenosis

## Abstract

**Background:**

The literature regarding the global cerebral blood flow (CBF) in idiopathic intracranial hypertension (IIH) is divergent leading to skepticism about the significance of blood flow to the disease’s underlying pathophysiology.

**Methods:**

The purpose of the current paper is to perform a PRISMA scoping review of the literature describing the CBF in IIH. The review investigated the PUBMED and Scopus data bases looking at case mix, technique and the methodologies employed by the studies selected.

**Discussion:**

Many studies indicate that the flow in IIH is normal but others show the flow to be altered. These later studies show a range of flows from a reduction of 20% to an increase of 50% compared to control values. Obesity is a common finding in IIH and is known to reduce CBF, anemia occurs in approximately 20% of IIH patients and is a potent cause of an increased CBF. Thus, variations in case mix may have a significant effect on the final outcome in those studies which are underpowered. The varying techniques which have been used to estimate CBF have differing strengths and weaknesses which may also have a bearing on the outcome. Some papers have significant confounding methodological issues.

**Conclusions:**

This review suggests each of the variables investigated are responsible for the divergent CBF findings in IIH.

## Introduction

The clinical syndrome of idiopathic intracranial hypertension (IIH), also known as pseudotumor cerebri, occurs in patients who present with high pressure type headaches and/or papilledema and visual obscuration [1]. At lumbar puncture, cerebrospinal (CSF) pressures are elevated but the CSF composition is normal [1]. The accepted cutoff for the diagnosis of IIH using the CSF pressure elevation is 25 cm H_2_O in adults [2]. Cerebral arterial inflow measured at the skull base varies with age. There is a rapid increase in flow from 7 months of age, with an average value of 800 ml/min, to a peak of 1200 mls/min at age 6 years [3]. Then there is a rapid decline until the age of 18 years of 51.6 ml/year to an average value of 750 mls/min [3]. From 18 years until 60 years, the decline is less rapid at 4.8 ml/year giving an ultimate value of about 600 ml/min [3]. General anesthesia can significantly reduce the cerebral blood flow [4]. The current author has published three papers utilizing phase contrast MRI imaging, measuring the arterial inflow in adults with IIH [5–7] and a single paper measuring the CBF in children being investigated for IIH [8]. In these papers there was strict matching of the controls for age and any anesthesia usage. In the first paper 12 patients with a secondary cause of intracranial hypertension i.e. sinus thrombosis, showed no significant change in arterial blood inflow compared to controls [5]. In the second paper, 21 patients with IIH had venous outflow sinus stenosis and showed an arterial inflow increase of 22% [6]. In the third paper, IIH patients, with fully patent sinuses, showed a 56% increase in arterial inflow compared to controls [7]. In the pediatric paper, patients thought to be at risk for the diagnosis of IIH showed a 34% increase in CBF compared to controls [8]. Despite these findings, there has been little interest in the possibility that an overall increase in blood flow underlies the physiology of IIH. This is because the literature is quite divergent regarding CBF in this disease. An initial review of the first pass indicator techniques used to estimate CBF in IIH suggested a range of findings, from 13% above normal to 19% below normal [9]. A paper using a Xenon^133^ washout technique suggested a 49% increase in CBF in this disease [10]. It is no surprise then, that review articles tend to suggest cerebral hyperemia in IIH cannot be confirmed [11]. These discrepancies within the literature have never been fully addressed. These preliminary findings suggest there may be significant gaps in the knowledge base surrounding CBF and IIH and a scoping review is the most appropriate way to search for the cause of these discrepancies [12].

The purpose of this paper is to perform a PRISMA scoping review to locate the available papers which have published quantified cerebral blood flow or inflow volumes, incorporating the majority of the brain volume, in either ml/100 g/min or ml/min in IIH patients. The inclusion criteria for IIH, the technique employed and the methodology selected will be collated. Once these studies have been obtained, the causes of the variability and discrepancies found between them will be investigated and discussed.

## Materials and methods

**Fig. 1:**
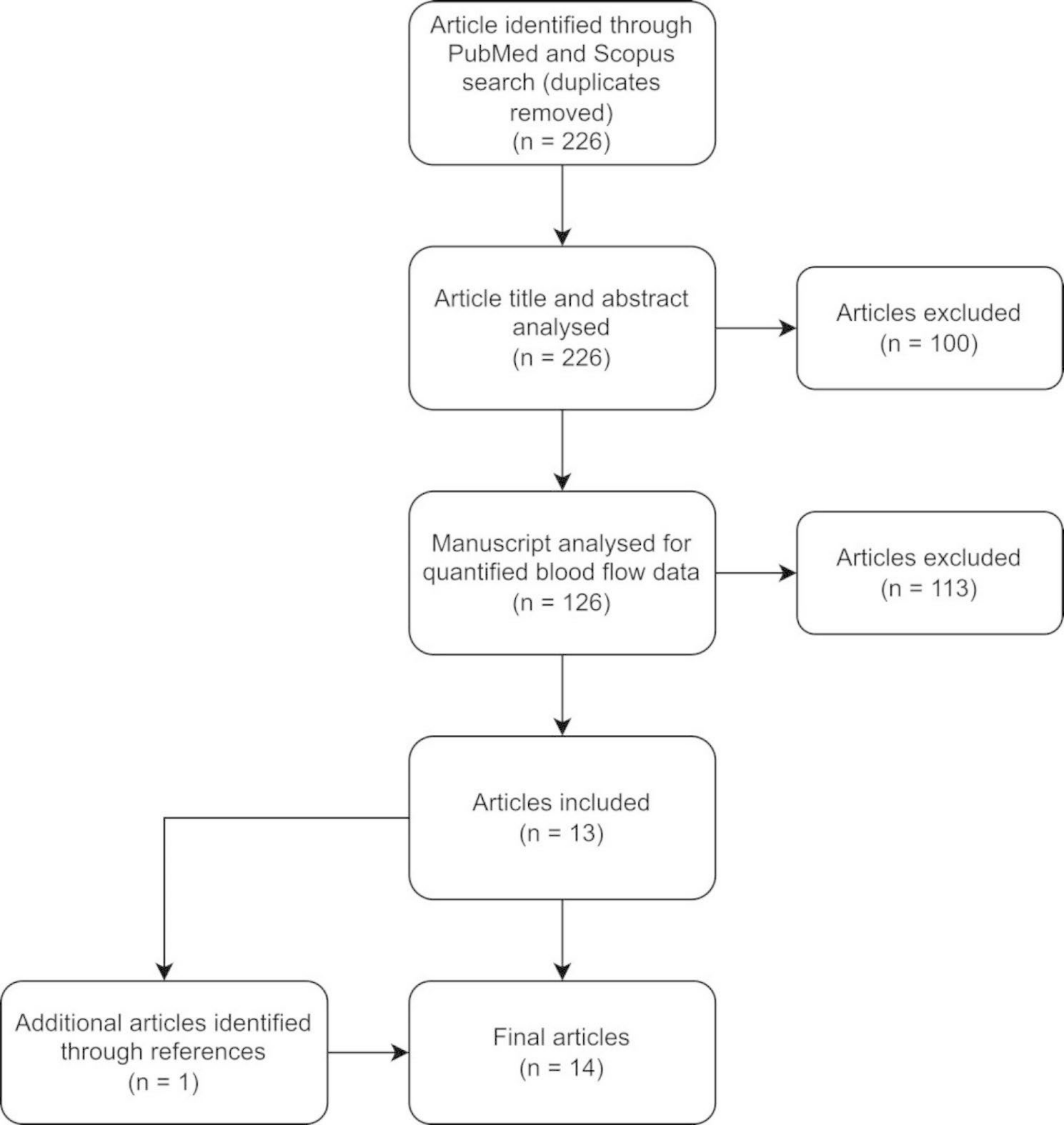
PRISMA flow chart. A chart showing the numbers of papers included and excluded at each stage

A scoping review was conducted with the application of the Preferred Reporting Items for Systematic Reviews and Meta-Analyses (PRISMA) criteria, to achieve the aims of mapping the available evidence and the knowledge gaps in the CBF measurement of IIH [12]. An open date method ending at June 2023 was utilized. Available studies in human subjects written in the English language were selected. The advanced search functions of both Scopus and PubMed were used to conduct the literature search of the respective databases. The query strings used for the search were: (idiopathic intracranial hypertension [Title or Abstract]) AND (blood flow OR arterial inflow OR blood velocity [Title or Abstract]). The specification of searching the title or abstract was included to restrict the total number of articles that were obtained in the search and to improve its precision by only including studies that were more likely to be relevant to the review. Figure 1 shows the method which was employed to determine the journal articles which were used in the final review. The query string was used to identify articles through PubMed and Scopus, with duplicates subsequently removed (*n* = 226). The title and abstract of these articles were then analysed. A total of *n* = 100 articles were excluded at this stage. Single case studies (*n* = 21) were excluded due to the inadequate sample size. Reviews (*n* = 28) were excluded, due to being comprised of multiple articles (their references were however, reviewed to look for relevant studies). Hypotheses, opinion pieces, commentaries, letters and correspondences were also excluded (*n* = 16). Animal modelling studies (*n* = 21) were removed. Finally, transcranial Doppler ultrasound studies (*n* = 14) were removed because (1) they only estimated flow in the middle cerebral artery territory (missing half of the brain volume) and (2) although arterial velocities were measured, the cross-sectional area of the vessels were not, so the volume flow rate could not be determined. The remaining (n = 126) were then reviewed looking at the entirety of the manuscripts. Articles not investigating idiopathic intracranial hypertension (n = 57) were removed. An investigation was then performed to find quantified cerebral blood flow data in either ml/100 g/min or ml/min for the majority of the brain volume from the remainder of the papers. Studies were excluded if there was no quantified flow data quoted, either in words or figures or if a minority of the brain was studied (*n* = 56). The remaining articles were included in the final review (*n* = 13). The references of the included studies and review articles were screened to assess if any of the referenced articles fit the inclusion criteria, of which one study was subsequently added to the final review (*n* = 14). The data items charted were the authors and publication date, the criteria for inclusion and the numbers of the patients and controls selected, the age of the patients/ controls, the percentage increase/decrease in CBF compared to the controls or the other criteria used by the authors and any confounders discovered in either the technique or methodology.

## Results

The 14 papers [6, 7, 10, 13–23] selected for further review are summarized in Table 1. The criteria used to diagnose IIH, the number of controls, the technique utilized and any confounding methodologies are summarized. There were 3 non controlled case series and 11 case controlled cross-sectional studies. There were 4 papers using first pass indicator techniques, 4 using indicator washout techniques, 5 using MR phase contrast technique and one using computational fluid dynamics calculation. Of the papers selected for review, there were three where the CBF was reduced by up to 20%, 5 in which it was normal and 6 in which it was increased by up to 50%.

**Table 1:** Papers for review

Authors	date	technique	patients / criteria	Age years	controls	CBF	confounders
Foley [13]	1955	N_2_O washout	3 IIH	19,36,45 (35±11)	10	52%	
**Gado et al.** [14]	**1975**	**H**_**2**_^**15**^ **O first pass**	**7 IIH**	**12-73**	**-**	**-20%**	**no controls, underestimation of higher flows**
Mathew et al. [15]	1975	Xe^133^ washout	2 IIH	23,32	-	-10%	no controls
**Raichle et al.** [16]	**1978**	**H**_**2**_^**15**^ **O first pass**	**10 IIH**	**17-45**	**-**	**-19%**	**no controls, underestimation of higher flows**
**Gjerris et al.** [17]	**1985**	**Xe** ^**133**^ **washout**	**14 IIH**	**34±15** **(43)**	**10**	**Normal**	**35% of patients ICP below 25 cmH**_**2**_ **0**
**Brooks et al.** [18]	**1985**	**C** ^**15**^ **O**_**2**_ **first pass**	**5 IIH**	**27±1** **(22-39)**	**15**	**Normal**	**IIH CO**_**2**_ **5.3 mmHg less than controls, underestimation of higher flows**
Gross et al. [10]	1990	Xe^133^ washout	9 IIH	34±7 (40±20)	26	49%	
**Bicakci et al.** [19]	**2006**	**MR Gd first pass**	**14 IIH**	**37±9** **(18-53)**	**14**	**Normal**	**all studies immediately after L.P., underestimation of higher flows**
Bateman [6]	2008	MR Phase contrast	21 IIH stenosis	29±14 (31±13)	14	22%	
Bateman et al. [7]	2009	MR Phase contrast	10 IIH patent 30 IIH stenosis	27±12 29±13 (31±13)	14	56% 23%	
Alperin et al. [20]	2013	MR Phase contrast	11 IIH obese	29±9 (31±9)	11	Normal	Obese patients only
Levitt et al. [21]	2016	MRV manometry CFD	6 IIH stenosis	N/A N/A	3	35%	Measured pressure/ stenosis and calculated flow
Capel et al. [22]	2018	MR Phase contrast	13 IIH	31±11 (37±7)	16	Normal	reported peak flow not mean flow, average pixel size 0.8 or 1.2 mm
**Juhasz et al.** [23]	**2018**	**MR Phase contrast**	**20 IIH**	**34±14** **(35±11)**	**20**	**Normal**	**pixel size 2.2 × 2.2 mm is too coarse**

Note.- C^15^O_2_, radiolabeled carbon dioxide; CFD, computational fluid dynamics; cmH_2_0, centimeters of water; CO_2_, carbon dioxide; Gd, gadolinium; H_2_^15^O, radiolabeled water; ICP, intracranial pressure; IIH, idiopathic intracranial hypertension; L.P., lumbar puncture; mm, millimeters; mmHg, millimeters of mercury; MRV, magnetic resonance venography; N/A, not available; N_2_O, nitrous oxide; SSS, superior sagittal sinus; Xe^133^, radiolabeled xenon. Control ages in brackets, Papers highlighted in **BOLD** have problems with the technique selected or methodological issues and are deemed uninterpretable

## Discussion

It has been argued that, on the basis of a normal pressure gradient between the CSF and sagittal sinus in IIH, the venous pressure, rather than an increase in CSF formation rate or increase in CSF outflow resistance, is the underlying cause of IIH [8]. Thus, the sagittal sinus pressure seems to be a major determinant of the elevated CSF pressure. However, an elevation of the venous pressure can occur due to a number of factors. There could be an elevation in the outflow pressure in the central veins of the chest, an increase in the venous outflow resistance secondary to venous stenosis or an increase in pressure secondary to cerebral hyperemia [8]. The absolute sagittal sinus pressure seems to be important in IIH and any elevation in the downstream pressure in either the transverse sinuses or the veins of the chest will increase the sagittal sinus pressure. Obesity occurs in up to 71% of patients with IIH [24] and obesity can increase the central venous pressure by up to 20 mmHg [25]. Therefore obesity will increase the sagittal sinus pressure. There are cases of obesity related IIH where bariatric surgery/ weight loss alone has induced cures [26, 27]. These findings would tend to suggest that there are some IIH patients with either isolated obesity or where obesity predominates as the cause of their disease. In young to middle age patients, obesity leads to a 12% reduction in CBF [28] so it would be expected that, in studies with a predominance of patients with obesity as the dominant feature, there could be a reduction in the overall CBF, especially in underpowered studies. In adults with IIH, up to 90% have been found to have a venous outflow stenosis within the transverse sinuses [29]. The exact quantification of a transverse sinus stenosis is difficult because the sinuses are triangular in cross-section and flow through a triangular tube is less efficient that a circular one [30]. In addition, the sinuses below the Torcular have parallel pathways. The normal anatomy could include one dominant right or left pathway only, or between 2 and 3 variously sized pathways. In order to be able to compare each of these variations directly, an equation giving an equivalent diameter of a single tube is required to reduce the complexity of the multiple tubes [30]. Despite these difficulties in stenosis quantification, in a large study, these stenoses have been shown to increase the venous pressure by up to 41 mmHg [31]. Treating these stenoses by stenting will cure some patients with IIH with no other therapy [31]. The later tends to suggest there may be patients with IIH who have outflow stenosis as the lone cause of their disease. As already discussed, secondary intracranial hypertension due to thrombosis does not appear to alter the arterial blood flow because the flow appears to be maintained by autoregulation, [5, 32] so IIH due purely to stenosis may be associated with a normal flow rate. Anemia has been shown to be associated with IIH in approximately 20% of cases [33, 34]. Anemia will increase the CBF by up to 5.5% per 10 g/L reduction in hemoglobin concentration [35]. In IIH associated with anemia, 73% of patients are non-obese [34]. In 60% of IIH patients with significant anemia, there is an improvement or resolution of their symptoms by treating the anemia alone [34] i.e. it is the lone cause. These final two points suggest that in some cohorts there could be an overabundance of anemic patients and so very high CBFs could be found. Thus, differing cohorts of IIH patients could have low, normal or elevated CBF depending on the cause of the underlying elevated venous pressure, so case mix will be important.

There are varying strengths and weaknesses of the techniques used to measure CBF in IIH, suggesting some of the variation could be due to technical factors. Finally, there are many possible confounding methodological issues which could also invalidate the findings of any one paper. Therefore, the purpose of this review is to discuss the IIH CBF literature looking at case mix, technical issues and confounding factors to try to make sense of the divergent findings in this disease.

### Case mix

**Fig. 2:**
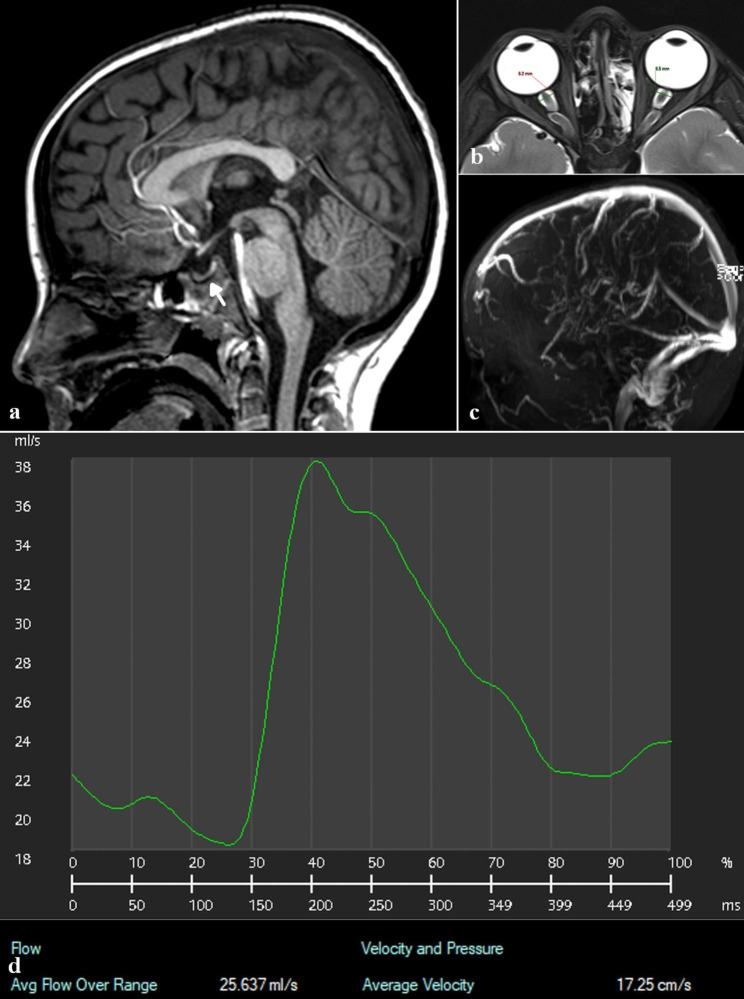
Elevated blood flow in a child with IIH. (**a**) A T1 image of a 6 year old male with IIH who was anemic. Note compression of the pituitary (arrow). (**b**) A T2 axial image through the orbits showing orbital flattening and dilated optic nerve sheaths. (**c**) A MR venogram showing no evidence of venous outflow stenosis. (**d**) A phase contrast flow graph of the arterial inflow at the skull base showing the inflow to be 25.6 mls/s or 1540 ml/min

It has been assumed that idiopathic intracranial hypertension is a single disease entity and therefore, should have a homogeneous underlying pathophysiology. As previously discussed, there are at least 3, and possibly more underlying disease processes leading to increased venous pressure which could be associated with differing CBF parameters. It has been discussed that anemia is associated with IIH and will increase the CBF in patients so affected. However, is there any direct evidence that anemia will increase the CBF in IIH? The answer is yes. In my most recent paediatric cohort, 42 children were being investigated for possible IIH [8]. A chart review of the hemoglobin concentrations of these children using the cut-off values for anemia as recommended by the World Health Organisation [36] found, of the 30 children who had hemoglobin concentrations contemporaneous with the MRI, 17% were anemic. This is similar to the incidence of anemia found in the IIH literature as already discussed. The 5 anemic patient’s hemoglobin concentrations averaged 113 ± 36 g/L. The average arterial inflow for these 5 children, as determined by phase contrast, was 1580 ± 440 ml/min vs. 990 ± 210 ml/min for the control group. The findings are illustrated in Fig. 2. This 6 year old male was status 8 months post bone marrow transplant for acute lymphoblastic leukaemia. He presented with headaches and vomiting. He was non-obese with a body mass index of 15 indicating no likely increase in central venous pressure. He was found to have papilledema at fundoscopy. He had a moderate normocytic anemia of 107 g/L. His brain MRI showed no structural abnormality but a compressed pituitary (see Fig. 1a). His globes were flattened and optic nerve sheathes dilated to greater to than 6 mm (see Fig. 1b). There was no venous outflow stenosis (see Fig. 1c) indicating no increase in venous outflow resistance or pressure at this level. The arterial inflow at the skull base was 25.6 ml/s or 1540 ml/min (56% higher than the controls and 30% higher than the expected figure for this age [3]) (see Fig. 1d). His CSF opening pressure was borderline at 26 cm H_2_O on L.P. whilst under anesthesia. His symptoms resolved and his fundoscopy returned to normal with improvement in his hemoglobin concentration and no other treatment. Anemia can significantly increase the venous pressure because the venous pressure scales with the square of the blood flow volume passing through the veins and not linearly [37]. The finding that 17% of my pediatric cases were anemic correlates with the 45% found to be hyperemic [8] (two standard deviations from the mean of the controls). This suggests there may be another factor involved in the cases who are hyperemic but not anemic. The study by Gross et al. may suggest a cause for this difference. Despite their patients initially having hemoglobin levels within the normal range, they noted a 28%, 31% and 23% drop in CBF in three patients who were followed up. These patients had an increase in haemoglobin of 14%, 8% and 17% compared to their initial study at follow-up respectively [10]. These changes were associated with reduced symptoms in these individuals [10]. Thus, an increase in the normal range haemoglobin level was associated with clinical improvement. This suggests there may be an increase in the sensitivity of the cerebral autoregulation to hypoxia in some IIH patients with elevated CBF despite a normal range hemoglobin.

**Fig. 3:**
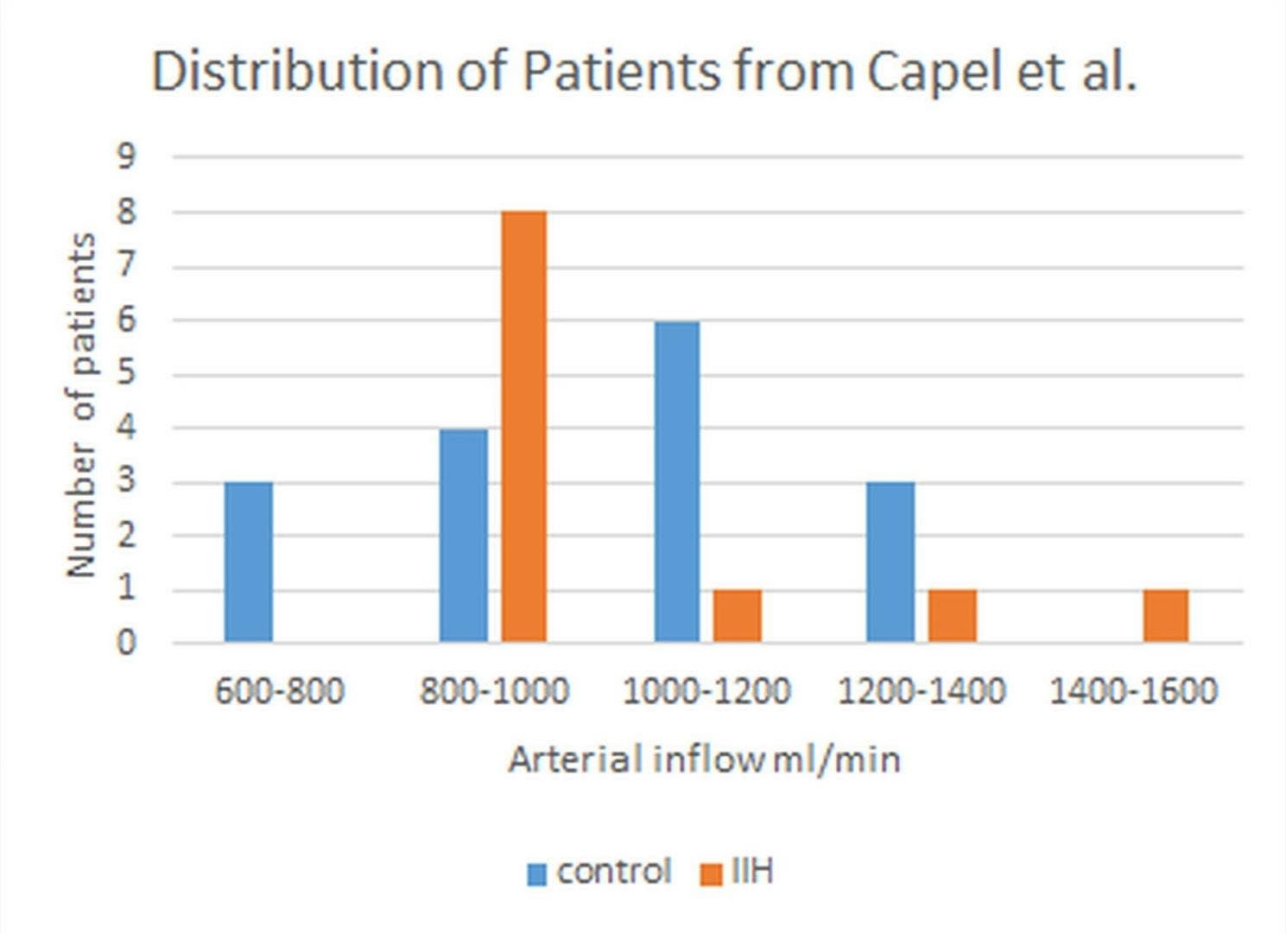
Distribution of patients and controls. A graph showing the distribution of patients in the study by Capel et al. [22], the control patients are consistent with a normal distribution but the IIH patients are skewed to the left with 2 outliers in the higher flow range

In half of the papers under review in Table 1, the number of patients entered was under 10. By chance we would expect 20% to have anemia i.e. 1 or 2 in each paper at most but with so few patients, the actual findings could be all anemic or none. This could largely explain the disparate findings in these underpowered papers. In the papers with larger numbers, it would be expected that there would be many patients with normal or low flow but a small number with high flow. Averaging them together will cancel out the effect and the final outcome will be no change in CBF compared to controls. However, the distribution in the cohort will appear skewed toward the lower flow rates with significant outliers in the higher flows. We can see this in two papers. In the first paper, Bicakci et al. [19] found the overall result was a normal CBF in IIH. However, of the 16 patients studied, there were 6 with flow two standard deviations below the mean compared to the controls. These were designated hypoperfused in the paper. There were 8 with flow in the normal range and two with flow greater than 8 standard deviations from the mean compared to the controls. The latter two were dismissed as just outliers and ignored. However, using the accumulative distribution function method [38] the probability that each of these 2 individuals could be this far from the mean for the controls is less than one chance in 10^15^ i.e. they have significantly increased flow. In the second paper, Capel et al. [22] measured arterial inflow in 13 patients and 16 controls. Unfortunately, only the peak systolic flow volumes and not the mean flow volumes were published, however, Dr Capel supplied the raw data to me for review (personal correspondence). The distribution is shown graphically in Fig. 3. When the control data was analysed with a Shapiro-Wilk test it was consistent with a normal distribution. The patient data, however, was skewed to the left (skew = 1.62). A Grubbs’ outlier test confirmed that the two patients to the right were outliers. Thus the data could be consistent with two populations, a larger one with slightly reduced CBF and two outlying patients with a larger CBF. Averaged together, the two groups show a normal CBF.

### Technical strengths and weaknesses

In 4 papers, the CBF was measured by using a first pass indicator technique, with two papers suggesting low flow and two normal flow. First pass techniques work by labelling the inflowing blood with a well-mixed tracer substance which is not metabolised by the brain [39]. The passage of the tracer is measured in both the arterial inflow and the brain tissue as a concentration vs. time curve [39]. The area under the curve is related to the cerebral blood volume (CBV) of the tissue. The comparison between the widths of the curve in the arterial inflow and tissue (allowing for mixing with the interstitial space using a partition coefficient) gives the mean transit time (MTT) [39]. Often considered the reference standard for flow estimation, H_2_^15^O imaging has a major weakness. It progressively underestimates the CBF at higher flows due to the limited time for water diffusion across the blood brain barrier (the partition coefficient is progressively less accurate) [40]. Butanol is a lipophilic agent with a high up take on first pass [41], which negates the problem of the tissue mixing. A study comparing butanol with H_2_^15^O showed a significant difference in flow estimation above 70 ml/100 g/min [41]. Unfortunately, this is not the only problem with these techniques. The theory of isotope indicator technique requires the passage of the indicator through the tissue to be homogenous i.e. all of the paths are equivalent [42]. Pseudo-Continuous Arterial Spin Labelling (PCASL) is an MRI indicator technique where the water in the arterial inflow is labelled by altering the T1 spins [43]. It is analogous to the other first pass techniques. Due to the short half-life of the spin labelling (1–2 s) in comparison with the known mean transit time of the brain (4–5 s), no signal should remain to be seen within the veins [43]. In high flow PCASL there is evidence of direct transit of labelled blood from the arterioles into the venules with signal seen within the venous sinuses implying direct arteriovenous shunting [43]. If all of the capillaries were equally affected then there would be a reduced efficiency of gas and nutrient exchange which would be counterproductive, suggesting there must be variable individual transit times. In sickle cell anaemia, venous signal on PCASL was noted in 6% of controls and 65% of anaemia patients, indicating higher capillary shunting with increased blood flow [44]. The effect of blood passing through two distinct pathways (the shunting path and the normal capillary pathway) at higher flows is to progressively artefactually widen the curve of the concentration time graph in the tissue and overestimate the mean transit time [45]. This is known as a bolus dispersion error [45]. As CBF = CBV/MTT [46] these techniques will greatly underestimate the CBF. Phase contrast MRI measurement of inflow does not vary in sensitivity with the flow rate [40]. Comparing PCASL and phase contrast MRI in anemic, hyperemic patients, phase contrast showed a 17% increase in flow but PCASL only a 9% increase (a relative 47% underestimation of PCASL at higher flow) [40]. Thus, as a group, first pass indicator techniques are not useful to measure CBF in an IIH cohort where a wide range of flow rates are expected because they will tend to overemphasize low flow and attenuate the high flow. For these reasons these techniques are listed as not being interpretable in Table 1.

In comparison to the first pass techniques, washout indicator techniques are less susceptible to the bolus dispersion error. In the later studies, a substance (usually a gas) is used which passes readily from the capillaries into the brain parenchyma. The brain is loaded till saturated and then the time activity curve is obtained following the washout phase. The time required for washout is in the order of minutes rather than seconds (as in first pass techniques) [39]. So there is no bolus dispersion error. Therefore, these techniques appear to be much more robust than first pass techniques. It is interesting to note in the review series, that the washout techniques show 2 papers where the flow is increased by approximately 50%, one where it is normal and one where it is reduced by 10% compared to no papers in the first pass indicator group where flow was increased.

Five of the papers for review utilised phase contrast MRI. Phase contrast works by placing all spins in a measurement plane in phase. A phase change is measured at a second time point by comparison with the first time point. Moving spins will alter their phase proportional to the velocity as they pass through the magnetic field. As the velocity and the cross-sectional area of the pixels is known, the flow rate can be derived by multiplication [39]. The addition of multiple pixels in a region of interest gives the flow rate. The papers by Bateman [6, 7] and Juhasz et al. [23] measured the carotid and basilar artery flow (supratentorial brain) and those by Alperin et al. [20] and Capel et al. [22] measured the carotid and both vertebral arteries thus adding the cerebellum to the flow to the brain. Despite this, a study simultaneously using both levels of acquisition showed no significant difference in flow measured between them [47] so this is unlikely to alter the results significantly. As already discussed, a strength of phase contrast imaging is that the estimation of flow tends to be unaffected by the rate of flow. This, however, only holds for pixel sizes of high enough resolution in comparison to the vessel being imaged. Due to partial voluming effects (where a pixel encompasses both flowing and non-flowing spins), the technique tends to overestimate the flow volume. However, the effect is stable and relatively limited provided the number of pixels per vessel diameter is greater than 4 [48]. The over-estimation is about 10% at 6 pixels per vessel diameter, 15% at 5 pixels per diameter and 17% at 4 pixels per diameter [48]. Below 4 pixels per diameter, the technique becomes less stable with overestimation peaking at about 40% at 3 pixels per diameter [48]. Between 3 and 2 pixels per diameter the overestimation drops to 25% and at 1.5 it is 10% [48]. As already discussed, the overestimation is relatively unchanged by the flow rate above 4 pixels per diameter. At higher flow rates there is increased luminal signal compared to the background tissue signal and the thus the signal ratio increases with higher flow rates [48]. Below 3 pixels per diameter the measurement error becomes a strong function of the signal ratio and is greatly increased as the flow rate increases. This is similar to the problem with the first pass indicator techniques. The average diameter of the internal carotid arteries and the vertebral arteries in 20–39 year olds is 4.8 ± 0.5 mm and 3.3 ± 0.3 mm respectively [49]. The basilar artery averages 3.4 ± 0.6 mm [50]. In my two studies, the pixel size was 0.7 mm indicating a stable homogenous overestimation of flow of about 10–15%, which would be acceptable. The study by Alperin et al. utilised 0.6 × 0.8 mm pixels [20], which would give a stable overestimation of 10–15%. The study by Capel et al. utilised 0.8 and 1.2 mm pixels, [22] the 0.8 pixel size gives an overestimation of 10–17%, the 1.2 mm pixel size gives an overestimation of 14% for the carotids but 40% for the vertebrals [48]. This last figure is high but possibly only affects a small number of measurements. Note, the blood flow for the controls for Capel et al.’s paper were 18% higher than in mine, indicating greater overestimation. These overestimations are probably acceptable providing they are compared with a group of well-matched controls. In the case of Juhasz et al. [23], the pixel size of 2.2 mm is completely inappropriate. This size maximises the variability of the overestimation due to the size of the vessel and also maximises the variability of the overestimation compared to the flow rate. The baseline carotid overestimation would be 30% (2.2 pixels/diameter) but the basilar overestimation would be less at 10% (1.5 pixels/diameter) or a threefold difference. A slight variability in the size of the carotid or vertebral vessels in any one individual would have a profound effect on the flow measurement returned. Due to this technical factor this paper has been designated uninterpretable in Table 1.

The final paper in Table 1 utilizes a technique where the flow volume was not directly measured but estimated. In the study by Levitt et al. [21] they used 3D MR venography data to construct an insilico model of the majority of the brain venous outflow in 6 patients with IIH. They utilised computational fluid dynamic modelling to estimate the flow rates required to generate the pressure drops they had previously measured. The pressures gradients were obtained using catheter manometry across the transverse sinus stenoses they had discovered. Comparing the three highest pressure gradient patients to the three lowest showed a 3 fold higher flow rate was required to generate the pressure found in the former as compared to the latter [21]. Comparing the average venous outflow volumes for all 6 of the patients to my control normal values for the same outflow volumes [6] indicated an overall 35% increase. A cohort of 158 IIH patients undergoing cerebral venography and manometery showed that non-invasive venography was an imperfect predictor of the pressure gradient [51]. The reason appears to be because there is a highly variable blood flow component which is not taken into consideration in the review of the stenosis severity alone. My IIH cohort supports this theory. In patients with thrombosed and almost occluded sagittal or transverse sinuses, the arterial inflow was normal [5]. In patients with a venous stenosis of the transverse sinuses greater than 70% the flow was increased by 19%, a 40–70% stenosis of the transverse sinuses required an increased flow of 28% to be symptomatic and when there was no stenosis, the flow was increased by 56% [7]. Thus, the stenosis grade and flow appear to interact to give the final sinus pressure and will affect whether the patient is likely to be symptomatic.

### Methodological issues

In three studies there were significant methodological issues which would compromise the findings. In the study by Gjerris et al. [17], 35% of the patients selected had a CSF pressure below the accepted cut-off level for IIH of 25 cmH_2_O indicating they would not qualify for the diagnosis. Under these circumstances the results of this study are unreliable and difficult to interpret. The study by Brooks et al. [18] showed that the expired partial pressure of carbon dioxide in the patients was 5.3 mmHg less than in the controls. This is indicative of patients on long term treatment with carbolic anhydrase inhibitors [52]. Indeed three of the 5 patients they studied were under long term treatment, raising doubts as to whether the patients still had the disease at all. For this reason the study is uninterpretable, however, it was already excluded because it was a first pass indicator study. The study by Bicakci et al. [19] has similar problems to the one by Gjerris et al. They studied patients within an hour of a diagnostic lumbar puncture. Because a lumbar puncture is both diagnostic and therapeutic in this disease it is likely the CSF pressures were normal at the time of the study, invalidating the results. Similar to Brooks et al., The study by Bicakci et al. was a first pass indicator study and was already excluded.

### Limitations

The review was limited to studies published in the English language, perhaps missing some relevant studies but encompasses an extensive time line. The specification of searching the title or abstract was included to restrict the total number of articles that were obtained in the search and to improve its precision by only including studies that were more likely to be relevant to the review, however, some studies may have been missed. Studies measuring blood flow in ml/min from single veins e.g. the superior sagittal sinus or the jugular veins but not including the vertebral veins or paravertebral venous plexus in the neck were excluded, as only a small percentage of the total brain flow would be captured. It is known that significant venous collateral flow exists in IIH [20] meaning this flow would be missed making these studies difficult to interpret. Similarly, studies using transcranial Doppler were excluded because only the middle cerebral arteries are sampled and thus exclude the majority of the brain. In addition, although the arterial velocity correlates with the blood flow volume, the correlation is relatively poor r = 0.42 [53]. A comparison between transcranial Doppler and xenon CT showed an error rate of for absolute flow estimation of transcranial Doppler of 18%, even when the blood flow changes were modest [54].

## Conclusions

Of the 14 studies selected for review, 6 were excluded due to either problems with the technique selected and or methodological issues with the way the studies were undertaken. Of the 8 remaining studies, in one the CBF was reduced by 10%, however, this is a study of only 2 individuals with no controls. The problem of case mix variability could account for this finding. There were two studies with a finding of a normal CBF overall, however, in one the patient distribution indicated some outliers with increased flow suggesting case mix was a significant problem. In the other normal flow study, Alperin et al., selected patients specifically to be obese [20] so normal to low blood flow is to be expected in this cohort. In the remaining 8 studies, the blood flow varied from 22 to 52% above normal. This would tend to suggest there are some patients with a component of elevated blood flow in IIH.

Given the difficulty of triaging patients to therapy on the basis of the severity of the venous stenosis alone (it may be absent in 10% of patients and does not correlate well with pressure), some form of blood flow determination is desirable. Most patients are already investigated with MRI. MR phase contrast flow measurement is fast, non-invasive and gives predictable results if the pixel size is carefully selected. Modern scanners can utilize pixel sizes between 0.5 and 0.6 mm with adequate signal to noise, suggesting this technique should be ideal for the purpose.

## Data Availability

All data generated or analysed during this study are available within the original papers or on reasonable request.

## Abbreviations

C^15^O_2_: radiolabeled carbon dioxide
CBF: cerebral blood flow
CFD: computational fluid dynamics
cmH_2_0: centimeters of water
CO_2_: carbon dioxide
CSF: cerebrospinal fluid
CBV: cerebral blood volume
Gd: gadolinium
g/L: grams per liter
H_2_^15^O: radiolabeled water
ICP: intracranial pressure
IIH: idiopathic intracranial hypertension
L.P.: lumbar puncture
ml/min: milliliters per minute
ml/s: milliliters per second
mm: millimeters
mmHg: millimeters of mercury
MRI: magnetic resonance imaging
MRV: magnetic resonance venography
MTT: mean transit time
N_2_O: nitrous oxide
PCASL: pseudocontinuous arterial spin labelling
PT: pulsatile tinnitus
SSS: superior sagittal sinus
Xe^133^: radiolabeled xenon
